# Supplementary material for: A Critical View of the Application of the APEX Software (Aqueous Photochemistry of Environmentally-Occurring Xenobiotics) to Predict Photoreaction Kinetics in Surface Freshwaters
Source: Molecules. 2019 Dec 18;25(1):9. doi: 10.3390/molecules25010009 (PMC7017383; doi:10.3390/molecules25010009)
Supplement: Supplementary file 1 [file molecules-25-00009-s001.zip › 3.2.4_gcc-4.4.0.zip!/3.2.4_gcc-4.4.0/3.2.4_gcc-4.4.0/doc/octave/html/interpreter/Utility-Functions.html]

Utility Functions - Untitled


Next: Special Functions,
Previous: Sums and Products,
Up: Arithmetic


---

### 17.5 Utility Functions

— Mapping Function: **ceil** (x)  
> Return the smallest integer not less than x. This is equivalent to
> rounding towards positive infinity. If x is
> complex, return `ceil (real (`x`)) + ceil (imag (`x`)) * I`.
>
> ```
>           ceil ([-2.7, 2.7])
>              =>  -2   3
> ```
>
>
>
>
> **See also:** floor, round, fix.

— Function File: **cross** (x, y)  
— Function File: **cross** (x, y, dim)  
> Compute the vector cross product of two 3-dimensional vectors
> x and y.
>
> ```
>           cross ([1,1,0], [0,1,1])
>                => [ 1; -1; 1 ]
> ```
>
> If x and y are matrices, the cross product is applied
> along the first dimension with 3 elements. The optional argument
> dim forces the cross product to be calculated along
> the specified dimension.
>
>
>
> **See also:** dot.

— Function File: d = **del2** (m)  
— Function File: d = **del2** (m, h)  
— Function File: d = **del2** (m, dx, dy, ...)  
> Calculate the discrete Laplace
> operator.
> For a 2-dimensional matrix m this is defined as
>
> ```
>                 1    / d^2            d^2         \
>           D  = --- * | ---  M(x,y) +  ---  M(x,y) |
>                 4    \ dx^2           dy^2        /
> ```
>
> For N-dimensional arrays the sum in parentheses is expanded to include second derivatives
> over the additional higher dimensions.
>
> The spacing between evaluation points may be defined by h, which is a
> scalar defining the equidistant spacing in all dimensions. Alternatively,
> the spacing in each dimension may be defined separately by dx, dy,
> etc. A scalar spacing argument defines equidistant spacing, whereas a vector
> argument can be used to specify variable spacing. The length of the spacing vectors
> must match the respective dimension of m. The default spacing value
> is 1.
>
> At least 3 data points are needed for each dimension. Boundary points are
> calculated from the linear extrapolation of interior points.
>
>
>
> **See also:** gradient, diff.

— Function File: p = **factor** (q)  
— Function File: [p, n] = **factor** (q)  
> Return prime factorization of q. That is, `prod (`p`)
> ==` q and every element of p is a prime number. If
> q `== 1`, returns 1.
>
> With two output arguments, return the unique primes p and
> their multiplicities. That is, `prod (`p `.^` n`) ==` q.
>
>
>
> **See also:** gcd, lcm.

— Function File: **factorial** (n)  
> Return the factorial of n where n is a positive integer. If
> n is a scalar, this is equivalent to `prod (1:`n`)`. For
> vector or matrix arguments, return the factorial of each element in the
> array. For non-integers see the generalized factorial function
> `gamma`.
>
>
>
> **See also:** prod, gamma.

— Mapping Function: **fix** (x)  
> Truncate fractional portion of x and return the integer portion. This
> is equivalent to rounding towards zero. If x is complex, return
> `fix (real (`x`)) + fix (imag (`x`)) * I`.
>
> ```
>           fix ([-2.7, 2.7])
>              => -2   2
> ```
>
>
>
>
> **See also:** ceil, floor, round.

— Mapping Function: **floor** (x)  
> Return the largest integer not greater than x. This is equivalent to
> rounding towards negative infinity. If x is
> complex, return `floor (real (`x`)) + floor (imag (`x`)) * I`.
>
> ```
>           floor ([-2.7, 2.7])
>                => -3   2
> ```
>
>
>
>
> **See also:** ceil, round, fix.

— Mapping Function: **fmod** (x, y)  
> Compute the floating point remainder of dividing x by y
> using the C library function `fmod`. The result has the same
> sign as x. If y is zero, the result is implementation-dependent.
>
>
>
> **See also:** mod, rem.

— Loadable Function: g = **gcd** (a)  
— Loadable Function: g = **gcd** (a1, a2, ...)  
— Loadable Function: [g, v1, ...] = **gcd** (a1, a2, ...)  
> Compute the greatest common divisor of the elements of a. If more
> than one argument is given all arguments must be the same size or scalar.
> In this case the greatest common divisor is calculated for each element
> individually. All elements must be integers. For example,
>
> ```
>           gcd ([15, 20])
>               =>  5
> ```
>
> and
>
> ```
>           gcd ([15, 9], [20, 18])
>               =>  5  9
> ```
>
> Optional return arguments v1, etc., contain integer vectors such
> that,
>
> ```
>           g = v1 .* a1 + v2 .* a2 + ...
> ```
>
> For backward compatibility with previous versions of this function, when
> all arguments are scalar, a single return argument v1 containing
> all of the values of v1, ... is acceptable.
>
>
>
> **See also:** lcm, factor.

— Function File: dx = **gradient** (m)  
— Function File: [dx, dy, dz, ...] = **gradient** (m)  
— Function File: [...] = **gradient** (m, s)  
— Function File: [...] = **gradient** (m, x, y, z, ...)  
— Function File: [...] = **gradient** (f, x0)  
— Function File: [...] = **gradient** (f, x0, s)  
— Function File: [...] = **gradient** (f, x0, x, y, ...)  
> Calculate the gradient of sampled data or a function. If m
> is a vector, calculate the one-dimensional gradient of m. If
> m is a matrix the gradient is calculated for each dimension.
>
> `[`dx`,` dy`] = gradient (`m`)` calculates the one
> dimensional gradient for x and y direction if m is a
> matrix. Additional return arguments can be use for multi-dimensional
> matrices.
>
> A constant spacing between two points can be provided by the
> s parameter. If s is a scalar, it is assumed to be the spacing
> for all dimensions.
> Otherwise, separate values of the spacing can be supplied by
> the x, ... arguments. Scalar values specify an equidistant spacing.
> Vector values for the x, ... arguments specify the coordinate for that
> dimension. The length must match their respective dimension of m.
>
> At boundary points a linear extrapolation is applied. Interior points
> are calculated with the first approximation of the numerical gradient
>
> ```
>           y'(i) = 1/(x(i+1)-x(i-1)) * (y(i-1)-y(i+1)).
> ```
>
> If the first argument f is a function handle, the gradient of the
> function at the points in x0 is approximated using central
> difference. For example, `gradient (@cos, 0)` approximates the
> gradient of the cosine function in the point x0 = 0. As with
> sampled data, the spacing values between the points from which the
> gradient is estimated can be set via the s or dx,
> dy, ... arguments. By default a spacing of 1 is used.
>
>
>
> **See also:** diff, del2.

— Built-in Function: **hypot** (x, y)  
> Compute the element-by-element square root of the sum of the squares of
> x and y. This is equivalent to
> `sqrt (`x`.^2 +` y`.^2)`, but calculated in a manner that
> avoids overflows for large values of x or y.

— Mapping Function: **lcm** (x)  
— Mapping Function: **lcm** (x, ...)  
> Compute the least common multiple of the elements of x, or
> of the list of all arguments. For example,
>
> ```
>           lcm (a1, ..., ak)
> ```
>
> is the same as
>
> ```
>           lcm ([a1, ..., ak]).
> ```
>
> All elements must be the same size or scalar.
>
>
>
> **See also:** factor, gcd.

— Function File: **list\_primes** (n)  
> List the first n primes. If n is unspecified, the first
> 25 primes are listed.
>
> The algorithm used is from page 218 of the TeXbook.
>
>
>
> **See also:** primes, isprime.

— Loadable Function: **max** (x)  
— Loadable Function: **max** (x, y)  
— Loadable Function: **max** (x, y, dim)  
— Loadable Function: [w, iw] = **max** (x)  
> For a vector argument, return the maximum value. For a matrix
> argument, return the maximum value from each column, as a row
> vector, or over the dimension dim if defined. For two matrices
> (or a matrix and scalar), return the pair-wise maximum.
> Thus,
>
> ```
>           max (max (x))
> ```
>
> returns the largest element of the matrix x, and
>
> ```
>           max (2:5, pi)
>               =>  3.1416  3.1416  4.0000  5.0000
> ```
>
> compares each element of the range `2:5` with `pi`, and
> returns a row vector of the maximum values.
>
> For complex arguments, the magnitude of the elements are used for
> comparison.
>
> If called with one input and two output arguments,
> `max` also returns the first index of the
> maximum value(s). Thus,
>
> ```
>           [x, ix] = max ([1, 3, 5, 2, 5])
>               =>  x = 5
>                   ix = 3
> ```
>
>
>
>
> **See also:** min, cummax, cummin.

— Loadable Function: **min** (x)  
— Loadable Function: **min** (x, y)  
— Loadable Function: **min** (x, y, dim)  
— Loadable Function: [w, iw] = **min** (x)  
> For a vector argument, return the minimum value. For a matrix
> argument, return the minimum value from each column, as a row
> vector, or over the dimension dim if defined. For two matrices
> (or a matrix and scalar), return the pair-wise minimum.
> Thus,
>
> ```
>           min (min (x))
> ```
>
> returns the smallest element of x, and
>
> ```
>           min (2:5, pi)
>               =>  2.0000  3.0000  3.1416  3.1416
> ```
>
> compares each element of the range `2:5` with `pi`, and
> returns a row vector of the minimum values.
>
> For complex arguments, the magnitude of the elements are used for
> comparison.
>
> If called with one input and two output arguments,
> `min` also returns the first index of the
> minimum value(s). Thus,
>
> ```
>           [x, ix] = min ([1, 3, 0, 2, 0])
>               =>  x = 0
>                   ix = 3
> ```
>
>
>
>
> **See also:** max, cummin, cummax.

— Loadable Function: **cummax** (x)  
— Loadable Function: **cummax** (x, dim)  
— Loadable Function: [w, iw] = **cummax** (x)  
> Return the cumulative maximum values along dimension dim. If dim
> is unspecified it defaults to column-wise operation. For example,
>
> ```
>           cummax ([1 3 2 6 4 5])
>               =>  1  3  3  6  6  6
> ```
>
> The call
>
> ```
>           [w, iw] = cummax (x, dim)
> ```
>
> is equivalent to the following code:
>
> ```
>           w = iw = zeros (size (x));
>           idxw = idxx = repmat ({':'}, 1, ndims (x));
>           for i = 1:size (x, dim)
>             idxw{dim} = i; idxx{dim} = 1:i;
>             [w(idxw{:}), iw(idxw{:})] = max(x(idxx{:}), [], dim);
>           endfor
> ```
>
> but computed in a much faster manner.
>
>
>
> **See also:** cummin, max, min.

— Loadable Function: **cummin** (x)  
— Loadable Function: **cummin** (x, dim)  
— Loadable Function: [w, iw] = **cummin** (x)  
> Return the cumulative minimum values along dimension dim. If dim
> is unspecified it defaults to column-wise operation. For example,
>
> ```
>           cummin ([5 4 6 2 3 1])
>               =>  5  4  4  2  2  1
> ```
>
> The call
>
> ```
>             [w, iw] = cummin (x, dim)
> ```
>
> is equivalent to the following code:
>
> ```
>           w = iw = zeros (size (x));
>           idxw = idxx = repmat ({':'}, 1, ndims (x));
>           for i = 1:size (x, dim)
>             idxw{dim} = i; idxx{dim} = 1:i;
>             [w(idxw{:}), iw(idxw{:})] = min(x(idxx{:}), [], dim);
>           endfor
> ```
>
> but computed in a much faster manner.
>
>
>
> **See also:** cummax, min, max.

— Mapping Function: **mod** (x, y)  
> Compute the modulo of x and y. Conceptually this is given by
>
> ```
>           x - y .* floor (x ./ y)
> ```
>
> and is written such that the correct modulus is returned for
> integer types. This function handles negative values correctly. That
> is, `mod (-1, 3)` is 2, not -1, as `rem (-1, 3)` returns.
> `mod (`x`, 0)` returns x.
>
> An error results if the dimensions of the arguments do not agree, or if
> either of the arguments is complex.
>
>
>
> **See also:** rem, fmod.

— Function File: **primes** (n)  
> Return all primes up to n.
>
> The algorithm used is the Sieve of Erastothenes.
>
> Note that if you need a specific number of primes you can use the
> fact the distance from one prime to the next is, on average,
> proportional to the logarithm of the prime. Integrating, one finds
> that there are about k primes less than
> k\*log(5\*k).
>
>
>
> **See also:** list\_primes, isprime.

— Mapping Function: **rem** (x, y)  
> Return the remainder of the division x `/` y, computed
> using the expression
>
> ```
>           x - y .* fix (x ./ y)
> ```
>
> An error message is printed if the dimensions of the arguments do not
> agree, or if either of the arguments is complex.
>
>
>
> **See also:** mod, fmod.

— Mapping Function: **round** (x)  
> Return the integer nearest to x. If x is complex, return
> `round (real (`x`)) + round (imag (`x`)) * I`.
>
> ```
>           round ([-2.7, 2.7])
>                => -3   3
> ```
>
>
>
>
> **See also:** ceil, floor, fix.

— Mapping Function: **roundb** (x)  
> Return the integer nearest to x. If there are two nearest
> integers, return the even one (banker's rounding). If x is complex,
> return `roundb (real (`x`)) + roundb (imag (`x`)) * I`.
>
>
>
> **See also:** round.

— Mapping Function: **sign** (x)  
> Compute the signum function, which is defined as
>
> ```
>                      -1, x < 0;
>           sign (x) =  0, x = 0;
>                       1, x > 0.
> ```
>
> For complex arguments, `sign` returns `x ./ abs (`x`)`.
